# On the Diseases of the United States’ Army on the Rio Grande

**Published:** 1848-06

**Authors:** William G. Proctor

**Affiliations:** Louisville, Ky.


### WESTERN JOURNAL

OF

### MEDICINE AND SURGERY.

JUNE, 1848.

Art. I.—*On the Diseases of the United States' Army on the Rio Grande.* By William G. Proctor, M.D., of Louisville, Ky. (An Inaugural Dissertation submitted to the Trustees and Medical Faculty of the University of Louisville for the Degree of Doctor of Medicine, March 8th, 1848.)

In the capacity of Hospital Steward to the Louisville Legion, I had an opportunity of observing every case of disease that occurred among its men. together with a great number in other regiments, and being much favored in having a principal and an assistant surgeon for our corps, who were ever ready and willing to render me assistance, I flatter myself I obtained a clear and intimate knowledge of the subject of which I am about to write.

The Legion was mustered into service without inspection; the consequence of which was that many of its members were wholly unfit for the service they were entering.

Many chronic diseases, and constitutions destroyed by lives of dissipation, or else by nature or previous habitude too delicate for military hardships, were early found in our regiment. From the very commencement of the campaign, too, we had to encounter inclemencies of weather and unfortunate circumstances of physical condition, likely, not only to irritate and inflame diseases already lurking in the system and cause to appear those to which there was a predisposition, but to produce almost every variety of sickness, even in those in sound health and of robust constitution. At Algiers, opposite New Orleans, where we first encamped after leaving Louisville, for several days previous to our departure, our quarters were literally inundated, and upon leaving that point our men were transported to the Brazos in crowded vessels, illy provided with clothing; even that they had being saturated with water and pestilential with filth. From this time forth on the part of the common soldier, there was but little regard for personal cleanliness, to which fact is attributable much of the subsequent disease and its virulency. Nor in their other habits were many of the regiment more observant of their health. Intemperance, unwholesome food, and irregularity of diet, were other prolific sources of ill health. The water too on the Brazos was intolerable, impregnated as it was with salt, sulphur, and other impurities. An exposure by day to a tropical sun of heat 80° to 105° Fahrenheit, and by night to a great depression of atmospheric temperature, was likewise very unfavorable to us.

Before reaching Camargo we were encamped for sometime at a point about twenty miles above the mouth of the Rio Grande, on a plain of clay soil, about sixty feet above low water mark. Here the rainy season of the country fairly set in on us, and we had showers every day falling sometimes in torrents for days and nights together. Our tents were thin and inadequate to our protection, while the soil would not absorb the rain; so that dry apparel or quarters were things unknown to us. This state of things lasted through the months of June and July. I must say, however, that we were during this period less exposed to vicissitudes of temperature than subsequently, though the heat was excessive by day and little relieved by sea-breeze.

Camargo, situated 250 miles further up the Rio Grande, was our next camping ground. Before reaching this place, the rainy season had to a degree subsided; though heavy rains occasionally fell. As to elevation and soil, we were located at this place about as at the one we had just left. The forest growth along the Rio Grande is the muskeet, a stunted species of the locust, intermixed with ebony. At Camargo the heat was excessive, ranging between 80° and 110° Fahrenheit, while no sea-breeze mitigated its fervor. The water, too, was here, as well as at the previous camp, unpalatable and unwholesome. When about leaving this for Monterey about the middle of September, the changes of atmospheric temperature were becoming sudden and marked.

At Monterey we remained till the latter end of December. During and for several days after the battle at this place, the troops were subject to many privations, to fatigue and exposure. It rained during this time for 48 successive hours. We were here encamped in a very low bottom, fertile and abundantly watered by numerous springs flowing from almost every mound. Our camp was bounded on its south-east side by an extensive swamp, and many similar ones were in our immediate vicinity. The forest growth of live-oak, hickory, &c. of large size, was covered with the celebrated Spanish Moss. Here the days were almost insupportable from heat, the nights from cold. The latter end of December, a forced march was made to Saltillo, seventy miles distant, where we were exposed to still greater vicissitudes of atmospheric temperature, with less means of protection from their injurious effects.

Such is a brief history of our campaign so far as it bears on my subject; in the examination of which I have had a work of more length and labor than I had anticipated or designed.

In the selection of it I have been governed alone by the interest which the subject affords.

In what I am about to say I will be guided more by my own experience than by a reference to books; this probably will account, in part, for my unsystematic statements of symptoms, causes, treatment, &c. In stating the causes of diseases, I have confined myself to such as have come under my observation. In symptomatology, as well as in treatment, I have strictly adhered to the same general rule. In speaking of remedies, space compels me to speak only in general terms, and to avoid specification, which is at any rate worse than useless, and would have proved the great excluder of many general and important facts. Such remedies as are specified, are so from evident reasons.

### A SCHEDULE of the Diseases of the Louisville Legion during the Campaign of 1846 and 1847 on the Rio Grande.

### TAKEN SICK OR RECEIVED INTO HOSPITAL.

| Months. | Fevers. | | | | | | | Erupt. Fever | | Diseases of the organs connected with the Digestive System. | | | | | | | | | | | |
|---|---|---|---|---|---|---|---|---|---|---|---|---|---|---|---|---|---|---|---|---|---|
| | Febris Con. Communis | Feb. Int. Quotid. | Feb. Int, Tertian | Feb. Int. Quartan | Feb. Int. Remittens | Feb. Typhus | Feb. Typhus Icterodes | Erysipelas | Rubeola | Cholera Morbus | Cholera | Diarrhoea | Dysentera Acuta | Dysentera Chronica | Dyspepsia | Enteritis | Gastritis | Icterus | Hepatitis | Obstipatio | Tonsilitis |
| June, | 12 | 3 | 2 | | | | | | | 6 | | 178 | 26 | | | 2 | 4 | | | 24 | |
| July, | 36 | 10 | 6 | | | | | 1 | 1 | | | 166 | 50 | 17 | 3 | | | 1 | 1 | 41 | |
| August, | 101 | 15 | 7 | | 1 | | | | 1 | 1 | | 55 | 9 | | 1 | | 2 | | | 18 | 1 |
| Septemb'r | 23 | 12 | 8 | | | | | | | | | 20 | 9 | | | 1 | 1 | 2 | 2 | 9 | |
| October, | 44 | 78 | 31 | | | | | | 5 | | 4 | 27 | 1 | | | | 3 | 1 | | 8 | |
| Novemb'r | 12 | 52 | 85 | 2 | 5 | | 1 | 1 | 1 | | 1 | 24 | 10 | 6 | | | 2 | | 3 | 6 | |
| Decemb'r | 35 | 31 | 9 | 1 | 3 | | | | | | 1 | 9 | 20 | 7 | | 1 | 2 | | 1 | 3 | |
| January, | 3 | 41 | 18 | 1 | 2 | 1 | | | | | | 23 | 19 | 7 | | | | | 4 | 3 | 1 |
| February, | 3 | 8 | 14 | | 1 | | | 2 | | | 1 | 13 | 12 | 6 | | | 1 | 2 | 1 | 1 | 1 |
| March, | 3 | 5 | 12 | 1 | 1 | | | | | | | 12 | 20 | 1 | | | 2 | 1 | | 5 | |
| April, | 6 | 6 | 10 | | 1 | 1 | | 1 | 1 | | | 10 | 6 | 8 | | | 1 | | | 1 | |

### *A SCHEDULE of the Diseases of the Louisville Legion during the Campaign of* 1846 *and* 1847 *on the Rio Grande*.

### TAKEN SICK OR RECEIVED INTO HOSPITAL.

Common continued or simple, fever as the first on our list, commands briefly our first attention.

Fever is essentially a disease of the whole system, for abundant evidence of which it is only necessary strictly to watch and carefully analyze its symptoms in a single case.

That it effects the brain is evident in the head-ache—delirium—coma—altered functions of the external senses, which may be either by perversion, or by increased or diminished activity. That it affects the nervous system generally, is evident from the general and indescribable uneasiness and soreness, suffered by every patient laboring under this disease. This is also exemplified in a most satisfactory manner in typhoid fever, in which the collapse forms so important a feature; the anxious and labored respiration of febrile patients is also manifestly dependent upon nervous derangement. That the intestinal canal is very powerfully affected, is manifest from the phenomena in the stomach, such as nausea and vomiting, flatulence, soreness, heat, &c., as also from the almost universal constipation of the bowels in every attack of this disease; and it is even asserted by some, that the stomach is so powerful a protector of the general system, that unless its functions be previously destroyed and its resistance subdued no febrile disease can be formed, and that, in its passage from local to general fever must pass through that organ and generate derangement there as a necessary link in its chain of phenomena. That the muscular system is affected, is evident from the great soreness and indisposition to muscular exercise. That the glandular system is affected to a very great extent is manifest from the first action of the disease never failing to diminish, to a great extent, the secretions in every part of the body. The liver ceases to secrete bile—the kidneys, urine—the salivary glands, saliva—the skin, perspiration; and even ulcers, pro tempore glands, secrete a diminished quantity of their ordinary discharges. The stomach also is impaired in its secretions, which properly accounts for the nausea, vomiting, distaste for food, &c., with the many other morbid phenomena presented in that organ. That the absorbents have their share also, is evident from the rapid emaciation, the absorption of hydropic extravasations, &c. That the heart and arteries are also concerned is evident from the excitement manifested in them; the pulse is all over the world considered one of the greatest, if not the greatest diagnostic in our profession—the frequency of the pulse is dependent entirely upon the heart's action; its other indications as hard, soft, wiry, corded, &c., are dependent either entirely on the state of the arteries or the conjoined action of the heart and arteries. May not the inefficient action of the heart depend upon the resistance the blood finds in passing over the arteries comparatively dry from a cessation of secretion in the mucous membrane?

These seem to me sufficient evidences of the truth of the position that fever is a disease of the whole system, and having enumerated most of the symptoms in evidence in the premises, we are now prepared to say a few words on its *treatment.*

The first indication in this, as in all other diseases, is to discover and remove, if possible, the exciting cause; and since the most common by far of these is found in the stomach or intestinal canal, a most careful investigation of these parts should always be instituted; and if the cause be found in these parts unaccompanied by lesion of structure, or too much inflammation, a full emetic of ipecac and tartar followed by a a cathartic, having its base in some of the mercurial preparations, will in many cases prove adequate. Nor is the efficacy of these remedies limited to cases dependent for their origin on disorders in the alimentary canal; for if they be not the first cause of the fever they are generated by it; and their removal is none the less indicated; hence we are led to consider them as important remedies in fevers dependent upon almost any cause. Nor is the curative agency of these remedies limited to this removal of the irritating substance from the bowels, but also by the almost specific effects of many of their ingredients upon the different parts of the system. The specific effects of tartar emetic in controlling the heart's action and in restoring cutaneous as well as other secretions are too well known to demand even a passing notice. So also the action of the base of the cathartic in producing its well known altertive effects. And that these secretions should be re-established is no less an item in the curation of fever than the removal of the exciting cause, for so long as these secretions remain arrested, so long will there be sufficient cause for fever. I should also have mentioned diluents which exert their influence by weakening and dissolving the morbid secretions as well as by their more direct action in relieving the abnormal temperature.

I must here caution my young friends in the profession upon the use of blood-letting. Whilst this is unequivocally the first remedy in all phlogistic disorders, it is as emphatically one which should be only used with the greatest caution. Here the young practitioner needs to be thus cautioned; all-powerful agents produce powerful effects, and whilst in experienced hands this remedy is probably productive of as much good as half the other remedies combined, in the hands of the inexperienced it is probably productive of as much injury as half of all the other remedies that can be used. Nor is this at all to be wondered at: he commences his career fresh from the halls of an university, where from an infinity to be taught, and the short space of time allowed for that purpose, the professor is compelled to deal only in general principles. These teach him that bloodletting is the most speedy and certain controller of inflammation, but it is utterly impossible for him to learn, without practical experience, the particular cases in which the remedy should be used or dispensed with. No professor, however great his capacity and experience, can lay down any universal and unvarying rules, which are to govern us in bloodletting, and no student, howsoever great his capacity, can learn, in a lecture room, all such indications. These are practical points and must be learned at the bed side. The young practitioner is too apt to bleed at any rate; he is too officious in the use of instruments, and is over anxious for the speedy recovery of his patients, and he not unfrequently bleeds his patient when he is not absolutely certain that it is demanded. And what are some of the evil consequences?

Why they are either prostration from which his patient never recovers, or at best a long and protracted convalescence which is bad enough in all conscience.

I could not, did even time and space permit, enumerate the various remedies which are considered valuable in this disease, and if I could it would be of no avail, since it would yet be more difficult to enumerate the particular crisis in the disease at which they should be administered: these are things as varying as the constitutions and idiosyncrasies of men, and as numerous as the circumstances under which they are placed. These, too, are things to be decided on only at the bed side, and he may consider himself well remunerated, who, after much experience and trouble, has acquired only a tolerable knowledge of these points.

But I cannot dismiss this subject without expressing the high regard I have for the tepid bath in diseases of this sort—or if the bath cannot be procured, for the sponging of the body with a sponge and tepid water. I have witnessed this remedy followed by so much success that it seems to hold a front rank among the anti-febrile remedies. I would be ungrateful to the remedy itself, were I not to mention it here, believing as I do, that to its agency I am indebted for, if not the lives, at any rate for the mitigation of much suffering in some of my best friends. Dr. C. had for several days been laboring under a sharp attack of fever, much prostrated, slightly delirious, or rather what we term *flighty*. Medicines refused to operate, venesection was attended by but temporary and slight relief; he was sponged cap-a-pie with tepid water; cutaneous action soon set up, a free alvine evacuation shortly followed, and from this time his convalescence was uninterrupted. I. G. had been for many days laboring under an attack which had resisted all treatment. His recovery had been entirely despaired of; typhoid symptoms had existed for several days; then coma; gums, tongue and teeth covered with sordes; and in fine every symptom of approaching dissolution. As a last respect to his sufferings, rather than a hope for his recovery, this kind of sponging was resorted to, and from this time may be dated his convalescence, and though it progressed very slowly we had the pleasure of seeing him entirely restored.

I shall now offer a few general remarks on intermittent fever, applicable to all its types, the tertian, quotidian, quartan, and all the extensions and complications of these primary types. The lassitude, pale and shrunken skin, small and frequent pulse, rigors, confused and inattentive mind, in violent attacks coma or delirium, pale urine, quick and anxious respiration, great thirst, &c. of the cold stage; nausea and vomiting, hot dry skin, full, strong and frequent pulse, free and regular respiration, high colored and scanty urine, headache, &c. of the hot stage; and the profuse perspiration, soft and full pulse, strong and frequent respiration, copious and sedimentous urine,, and in fine, a general abatement of all the symptoms of the preceding stages until it terminates in apyrexia of the sweating stage: These are symptoms, I repeat, applicable to all its forms except that occasionally, though very rarely, we meet with an anomalous case, such, for instance, as an apparent entire want of the cold stage.

In remarking on this disease, I shall consider it essentially and strictly a nervous disorder. So near is it allied to other nervous affections, and so inseparable are they, that I can but consider them sisters of the same genera. To this I have come rather from an examination and analysis of the symptoms than from the teachings of books or my kind preceptor to whom I am indebted for the warning more than once repeated of "*beware of your nervous irritation.*" But let us compare for a moment the symptoms, causes, and treatment of this disease with those of other nervous affections. Like other nervous diseases it finds its favored victims in pale asthenic persons—persons who live on a scanty and insufficiently nutritious diet, in such as never had any constitutions at all, or whose constitutions had been previously destroyed by disease or dissipation, in those much exposed to vicissitudes of temperature, and above all, in inhabitants of low, damp, marshy countries. Like other nervous disorders, it is remitting or intermitting in its action. And again, all the remedies used in the cure of this disease are addressed to the nervous system. Quinine, iron, bitters of various sorts, &c., are, it seems to me, addressed solely to the nervous system, strengthening that portion of the economy, and thereby producing their effects upon the disease. I am aware that the doctrine is now held by more experienced persons than myself, that quinine as the representative of bark does not produce its effects by its tonic properties, but by a "*specific action upon the poison inducing the disease;*" now that it does act specifically, if you please, upon the poison I am willing to confess, but I do hold that this specific action is produced by a secondary process, that is, through the nervous system by its tonic properties. No one pretends to claim for all the tonics *alterative properties*, yet they cure intermittent fever, and it seems to me upon the very same principle as quinine. I cannot enter into a further discussion on the modus operandi of medicines. It is a subject I have carefully avoided as being one of as much evil as good, since it does not in any manner promote the action of remedies, and serves only to embarrass the young student.

Having taken the ground that the predisposing cause of intermittent fever proper is in the nervous system, let us next consider some of the most frequent exciting causes. Among these, first, I will mention

*Malaria*, not only because it is almost universally considered the great source of this disease, as well by the people as by the profession, but also because experience has shown me that all other things being equal, we had more than *five times* the number of cases of this fever whilst situated in malarious localities and under circumstances favorable to its influences. For the evidence of which I have only to refer to the accompanying schedule and prefatory statements. Now it must be borne in mind that with the exception of position, we were subject to almost the same causes of disease throughout the campaign; that they were the same constitutions, living on the same food, subjected to the same privations, and exposed to the same vicissitudes of temperature. With these facts before me, I can come to no other conclusion than the proposition with which I started out in this sentence—that malaria is the most prolific progenitor of intermittent fever. Now as to the manner in which malaria produces its effects, I can say nothing, unless it be through the vast expanse of nervous tissue plated out on the surface of the body and on its continuation, the mucous membrane of air passages and lungs. This seems abundant surface to expose to its action. The mode in which the debility is produced, is, probably, by its first action upon the nervous extremities, and through them upon the nervous centers, thereby deranging the entire healthy process, causing the formation of unhealthy blood, which in its turn nourishes the parent which produced it. This seems also, to account for the appearance of intermittent fever in persons long subsequent to their exposure to malaria. As collateral evidence that it acts upon the skin, flannel worn next the skin, proved with us one of the best prophylactics to this as well as to other diseases which might be ascribed to the same causes.

*Vicissitudes of temperature* seemed to be the next most prolific exciting cause, and to this single one I am inclined to ascribe most of the cases of this disease arising in low malarious positions. Nor is it at all strange, if I am correct in the opinion that this is a very prolific parent of this disease, that we were, when most removed from miasmatic localities, subject to no inconsiderable amount of intermittent fever, when it is borne in mind that we experienced the most sudden changes of temperature, as much as 50° to 60° in a very short time, connected with the fact that the men were very poorly provided with clothing and other protection, and exhibited great recklessness as regards their habits.

These, without enumerating others, seem to my mind abundant causes for the explanation of the amount of intermittent fever to which the regiment was subjected, and also satisfactory evidence on the long contested points, as to the question of the production of intermittent fever.

In the preceding remarks I have included none but intermittent fever proper, or in other words, that kind of intermittent fever which is not ascribable to any discoverable pathological state of the system.

On the treatment of intermittent fever a very few words will suffice. The antidote is in the mouth of every one. Who does not know that quinine is an almost infallible cure for intermittent fever? As regards the administration of this remedy, it should be measured by effects and not by grains. I have rarely seen '*heroic*' doses, given two or three hours before a paroxysm, fail in arresting it. Its action should be kept up in the system by the administration of smaller doses at short intervals, that the system may be kept continually under its effects, which should also be continued a short time after the disappearance of the disease. The clearing out of the stomach and intestinal canal with a free emetic and cathartic should under ordinary circumstances be generally practiced; it leaves the system much more susceptible to the action of the subsequent treatment; nor do I know of a more speedy and effective means of hastening on the sweating stage than an emetic given in either of the preceding.

The various preparations of *iron* are in high repute in the cure of this disease. I have tested its efficacy in form of carbonate, on poor Mexicans, whose indigent circumstances and the enormous price of the quinine prevented their use of it; it never failed to produce the desired effects in any case in which I have seen it administered—notwitstanding I have seen it tried under the most inauspicious circumstances, in persons resident in malarious districts, and who have been subject to the disease for many years. An infusion of a kind of willow is much used by the poorer Mexicans and with very happy results. A doctor and an apothecary in Saltillo informed me that they rarely used any other remedy, and that its administration was crowned with the happiest results. I suppose its curative properties are dependent upon the salicine which it contains. Arsenic is also a precious remedy in this disease. I have seen much good follow upon its administration. Fowler's solution in combination with tincture of opium, commencing with small doses and increasing as the system becomes accustomed to it, is a very excellent preparation, and is probably less offensive to the stomach than the crude arsenic. Almost all the stimulants are occasionally used during the apyrexia of the disease. I have seen the paroxysm checked in some and postponed in others by the administration of a bolus of opium, camphor, and ammonia. Capsicum, in combination with many tonics and stimulants, and alone, is not unfrequently a very good remedy. Venesection is recommended on the high authority of Dr. Mackintosh; this is certainly very valuable as a palliative remedy, it being the most speedy means of hastening the cold and hot stages. It should only be carried to a sufficient extent to restore the equilibrium between the nervous and circulatory systems; if carried to too great an extent, it will be productive of most pernicious effects. I have only witnessed its use in a single case, and that was in private practice, and bloodletting was indicated from other reasons than for palliative purposes, there being manifest appoplectic tendency; the loss of a very small quantity of blood was followed by great amelioration of the symptoms and the bleeding was not carried farther.

Why would not electricity be a good remedy in this disease; it is a nervous remedy, and acts upon the nervous system—and intermittent fever is a nervous disease seated in the nervous system.

Next we have diseases of the nervous system, and in this groupe I include only such as are primarily founded in the nervous system, or, if dependent upon another disease, exhibiting themselves as an anomaly of the nervous system.

The proximate causes are morbid affections of organic life, disturbance of functions, &c. The predisposing causes, heat, cold, climate, exposure, moisture, malaria, idleness, loss of blood, masturbation, mental emotions, local and specific irritation, the presence of foreign substances, &c.

The first indication in the treatment of nervous diseases, like that in all others, is to search for the exciting cause; here lies the difficulty in their treatment, for as various as the exciting causes, are the indications of treatment. If it be found dependent upon a plethoric state of the system, then an antiphlogistic treatment is indicated. If upon an asthænic state of the system, then a tonic treatment is as surely indicated. If upon the presence of foreign substances the removal generally relieves the symptoms. In short, if the disease is idiopathically nervous, the treatment should be primarily addressed to the nervous system; if it depend upon any discoverable cause, then the treatment should first be addressed to the removal of that cause. I now remember several cases in which the symptoms were so obscure, that they baffled every effort to discover the cause; every means used for their relief was either fruitless, or if attended by any relief, it was only temporary. I will relate one. J. Early, a private in company I, had been troubled for a long time with "*sun pain*" over the right eye; the most careful examination could discover no other symptoms of the disease upon which to base the nervous disorder; still the most active nervines did not produce the desired effect. Early continued to complain of his sun pain, and being rather suspected of malingering, a full dose of the oil of turpentine was administered, which to the astonishment of all, produced the evacuation of several feet of *tape-worm.* The dose was repeated several times afterwards and a considerable quantity of tape-worm was discharged; after this time no more was heard of Early's "sun pain."

One of the most common causes of idiopathic neuralgia is nervous debility, and this is probably the indication to be treated in nine-tenths of the cases, for if it be not primarily dependent upon this state of things, it is speedily generated by it, and in its turn becomes the supporter of the disease and the indication to be treated. And since all partake more or less of periodicity combined, as I have above stated, with debility, the very best remedy that can be found is that which partakes at the same time of antiperiodic and tonic properties; as fulfilling these indications, the preparations of cinchona stand unrivalled. I do not now remember a single case of neuralgia or intermittent neuralgia, in which the administration of these remedies was indicated, which did not finally yield to them. A most stubborn one came under my observation in Captain Saunders. He had for a long time been subject to an intermitting neuralgia, which came on with renewed vigor after an exposure; he took quinine in enormous doses, as much as half an ounce per day for several successive days; the disease was finally checked and he had not had another attack when I last saw him, which was several months after the cure was effected.

Iron in its various preparations is highly recommended in these disorders—of its powers I have but little personal experience, though they would seem, from their effects, to be peculiarly adapted to this state of things; the sesquioxide is the preparation most highly recommended. Arsenic is highly spoken of. I have seen it administered in the form of Fowler's solution, but do not remember any marked beneficial results. Opium, strychnia, conium, stramonium, aconite, &c., are much recommended by some, but they are all remedies addressed rather to the symptoms than to the cause of the disease. I am persuaded that the endermic mode of application is the preferable—their effects are very speedily manifested when applied over the course of a painful nerve previously denuded of its epidermis by a vesicant. Various counter irritants are much in vogue, though they should be cautiously used, especially in subcutaneous nerves. In the deeper seated forms they are more applicable. I witnessed a case of sciatica last fall, which was almost immediately relieved by a blister. I have never seen the moxa, acupuncturation, ammonia, &c., used.

From the number of remedies which have been used in this disease, it is evidently one which not unfrequently baffles the most scientific; the vaguest superstitions and the most powerful remedies not unfrequently have shared similar fates; and I now repeat, that if the remedies above mentioned fail, we have but little left to rest our hopes upon.

Another group of nervous disorders, differing very widely from the preceding neuralgia or nervous irritation, is melancholia, a general disorder of the whole nervous system; of nostalgia, one of its varieties, I have now to speak.

Of the predisposing and exciting causes of nervous derangement enough has already been said, but I must here repeat, that previous habits, mental developments, great domesticity, mental and physical inertia, temperament, masturbation, &c., are probably the most common predisposing causes of this special form of these disorders; the exciting were generally previous diseases, disappointment, &c.

The treatment is preventive and palliative. I do not now remember a single recovery from this disease, after it had become thoroughly developed. The best preventive to this disease was exercise, mental or physical; the mind is most prone to melancholy when unoccupied by any sort of exertion. The disease was always most rife when we had been encamped for a considerable length of time in one position, and when the soldiers were permitted to remain in idleness: this disease was manifesting itself to a fearful extent in a company of young countrymen, who had been for several weeks encamped in one position and permitted to remain idle. A long march entirely destroyed the tendency, which did not recur until the same circumstances developed it.

Mental emotions had also great effect upon the disease. If the mind can be aroused into action, the disease may be considered cured. An example of this is shown in the following case:

T. B. F., a young man of sensitive feeling, had been for several days affected with the premonitory symptoms, great taciturnity, unless on matters pertaining to his returning home, disinclination to association, sadness, &c. On applying to the surgeon for a discharge, he was reprimanded in a caustic manner, which aroused his pride and anger; he left the tent, and from that time no more was heard of Mr. F.'s inability to do his duty as a soldier. This treatment will not answer in more advanced stages as was evident in the case of F. S. Stimulated by the happy result in the preceding case, the same treatment was tried in one more advanced, but this was as near proving fatal as the preceding had been fortunate; the patient sank as if stunned by electricity, and did not again arise from his bed until a discharge was procured for and presented to him. This seemed to act like a charm,—in half an hour he was making active preparations for his departure, and a few hours saw him cheerfully on his way home. I. B. upon receiving a final refusal upon an application to be discharged was seized with symptoms of palpitation of the heart, which terminated his life in a few hours.

The following case will probably present, as well as a more extended description, the rapidity and irremediability of the disease. J. P., a young man of good constitution and habits, was attacked with a tolerably violent diarrhœa, this yielded to the ordinary remedies. Melancholic symptoms soon running into nostalgia, supervened; he ceased to notice surrounding objects, lay apparently comatose, when aroused and stimulated to conversation he always spoke of home, held conversation with his relatives, muttered disconnected sentences generally about domestic affairs, such as calling dogs, driving hogs, talking to children, &c. There were present no other symptoms of ce rebral inflammation, no preternatural heat about the head, no flushing of the face; pulse soft, weak, and slow; the tongue was clean; and in short there was no indication for any sort of treatment, except the condition of the mind which I have already described. There was a relaxation of the sphincters for twenty-four hours previous to dissolution. Symptoms of cerebral inflammation are fequently seen; these demand blisters along the spine and the nape of the neck; this treatment was followed by good results in two or three cases in which inflammation of the brain was suspected. This disease is sometimes considered to depend entirely upon cerebral inflammation; if it does, the symptoms in the majority of cases are not to be discovered by the common practitioner.

Next on our list come diseases of the alimentary canal; nor is this by any means an unimportant group; in fact they outnumbered, for several months, all other diseases combined.

I shall say nothing on chronic gastritis or dyspepsia, nor acute gastritis, nor inflammation of the intestines. Chronic gastritis is a disease for which little can be done in the field, and of which, we fortunately had but little. Acute gastritis and inflammation of the bowels demand prompt attention and antiphlogistic treatment to its full extent; disorders of such important organs are not to be tampered with. Counter excitants, anodynes, and mercurials are precious remedies in this disease.

Diarrhœa made no unimportant part of the disease of our regiment. The principal causes of this disease were, unwholesome ingesta, excess of eating and drinking, exposure, heat, cold, moisture. The principal feature in this disease, is the increased peristaltic action; the minor symptoms are very numerous varying according to the exciting causes. That induced by over repletion of the stomach, or unwholesome ingesta, is generally characterised by a foul tongue, bad breath, nausea and flatulence. All diarrhœas should be primarily considered sanative processes, attempts of nature at removing offending causes; therefore, if there is no contra-indicating symptoms, they should be permitted to run their course; if anything is needed from the practitioner it is only to assist nature. A free emetic of ipecac will not unfrequently be the only remedy required, or a free purgative may answer the same end. But should the disease run on and continue to weaken the patient, then the indication is unquestionably to suppress the aqueous discharges; the best means for accomplishing this end is the use of some of the common astringent tinctures, which may be combined with a small quantity of tinct. opii. camphorata, or common tinct. opii. Acetate of lead is a valuable remedy in this disease, especially in combination with Dover's powder. The almost specific action of the ipecac and opium, in controlling fluxes from the body, bloody or serous, is too well known to demand any comment. Opium alone was frequently followed by excellent results, especially in cases in which there was fear of inducing or increasing gastric irritability. Caution is required in the administration of this all-powerful remedy, lest, by too speedily suppressing a diarrhœa, a more malignant disease be induced. A remarkable circumstance is, that this disease became more and more untractable, often refusing to yield to the milder remedies; each month demanded an increase of the size of the doses. Now I do not intend to convey the idea that this expectant treatment was the one generally adopted by us—such is not the case. So great was the tendency of this disease to run into dysentery, and a matter of so much importance was it, in an enemy's country, where we were at any moment liable to be called into active service, to preserve the strength of the soldier, that it was necessary to administer to the very first symptoms to prevent the supervention of inflammation, and also to prevent the rapid loss of strength occasioned by the serous discharges. Diarrhœa depending upon ulcerations, metastasis, &c., demands remedies addressed especially to such causes. In all cases of diarrhœa antagonism of the skin is to be attended to.

Dysentery, another of the same family, and by far the most important, is too often, by the inexperienced, confounded with diarrhœa, while in fact, scarcely any two diseases are more radically different, their only points of resemblance being that they are both affections of the alimentary canal, and are both characterized by frequent stools. Diarrhœa is a discharge of unnaturally thin faeces unaccompanied by fever; in dysentery there is but little or no fæcal matter and that which is evacuated is in small hard lumps, mucus—and a mixture of mucus and blood constituting the character of the discharge—and always more or less fever. Diarrhœa *may be* attended with griping and pain, whilst griping, tenesmus, and pain are universal symptoms in dysentery.

Among its most prolific sources with us were climate, season, vicissitudes of temperature, wet, exposure, unwholesome food, violent exercise, malaria, diarrhœa, and other intestinal diseases. It is useless for me to go into a description of the modus operandi of these various causes; every one knows that diarrhœa produces a predisposition to dysentery, by inviting an increased afflux of blood to the intestinal canal, and that the various other causes exert their influence in equally manifest and simple ways. As to malaria, I cannot say positively, whether or not it produces dysentery by its direct agency, though I believe it does. I have frequently seen dysentery alternate with intermittent fever or partake of a regular intermittent type, for which I am unable to account except by the direct agency of malaria. Again, statistics, as appears from our books, shew a vast increase in this disease when in malarious districts. That it produces dysentery, by its secondary effects, cannot be denied; any cause which engorges the internal organs, spleen and liver, will also engorge the lower intestines, and any cause which will engorge that portion of the intestines, will predispose to dysentery. The liver by being engorged has also to secrete a larger quantity of bile, the natural intestinal stimulus.

Dysentery is not a contagious disease so far as I have seen it; true it is, that it sometimes appears epidemic, but that must be ascribed to predisposing cause existing in the atmosphere at the time. I have seen, through the campaign, men affected with dysentery sleeping in the same tents, using the same blankets, and eating the same food as their messmates, yet the latter seemed no more prone to the disease than men who were free from any such proximity, or beyond any such other means of contagion.

The *symptoms* of dysentery are, fever, thirst in the first stages, hard and frequent pulse, hot skin, first furred and then a red and glazed tongue, accompanied with rapid diminution in the volume of the pulse; tenderness on pressure, acute pain subject to exacerbations and diminutions, very frequent and sometimes almost constant inclination to go to stool, with a sensation that something is just ready to be voided, tenesmus, griping occasionally, dysuria, &c. The post-mortem phenomena (if correctly reported, for of these I have no personal knowledge) are inflammation and frequently ulceration of the lower portion of the colon and rectum, thickening of the mucous membrane, morbid vascularity of the mesocolon, sometimes perforation by ulceration and sometimes mortification of the portion of the intestinal canal already indicated as the seat of the disease.

The symptoms and post-mortem phenomena of this disease shew it essentially an inflammatory disorder; the *treatment* is consequently antiphlogistic. If the case be of recent origin, and there is reason to believe that the exciting cause still remains in the stomach, (if it be dependent upon such cause) then the same indication and mode of removal as in diarrhœa, is probably the most speedy method of relieving it, to-wit: by an emetic; if it be in the intestinal canal and out of the reach of an emetic, a full cathartic will probably remove it. The administration of an anodyne and aperient alternately is a practice frequently followed by much success; an anodyne and aperient administered conjointly seem to produce their separate and distinct effects, without at all antagonizing. If aperients are demanded at all, as unquestionably they are, it is in the forming stage; the removal of all foreign matters from an inflamed surface is always demanded under all circumstances. I can safely say that this treatment is a good one from my own experience. The administration of mercury was with us the "*great treatment;*" so common was it that dysentery was connected with portal derangement that the rule, in every case, was to give mercury—the exception, not to use it. Blue mass combined with Dover's powder or even opium, was considered and acted almost like a specific.

How the disparity of opinion among authors, as to the use of mercury, could have arisen, I cannot see; certainly those who oppose its use never had to treat dysentery in a tropical climate. With us there were but few cases which demanded bloodletting, this probably depended upon the fact that treatment was generally instituted in the forming stage. Opium should be cautiously administered in the inflammatory stage; it is too apt to mask the more prominent symptoms and lull the practitioner as well as patient into a state of false security. Anodyne injections are very excellent remedies especially in that stage, in which, after the violence of the attack has been subdued, the tenesmus, pain, and strangury still exist. Care should be taken that there be not too much starch or mucilage of whatever sort, of which the vehicle is composed, lest its presence excite the act which it is intended to control. Counter irritation is a valuable remedy after the inflammatory stage is subdued; if applied during the height of the inflammation, blisters are rather inclined to propagate than control the inflammation. In chronic cases their use is indispensable.

The greatest attention should be paid to diet, which should be scanty and antiphlogistic; during convalescence also this is to be kept in mind, but it may be more nutritious. Antagonism of the skin is also an important consideration in the treatment of this as well as of other abdominal diseases. Clothing should be abundant and woollen next to the skin; this is a very valuable item—so valuable is it considered that many of the soldiers are permitted to wear nothing but flannel next the skin. The patient should preserve the recumbent posture; every exertion is followed by disagreeable symptoms. The tepid bath in the inflammatory stage is good treatment. Tonics are indicated under two circumstances: first, when there is a recent tendency with intermittent fever, or any tendency to intermittent symptoms. I have seen this disease assume a regularly intermitting type and obstinately resist all treatment until combined with quinine. They are, secondly, indicated when the system has been much prostrated by the disease, and is deficient in elasticity or recuperative energy.

A remedy which I have rarely seen fail, when almost all others had, is nitric acid gtt. viij. elixir vitr. ʒss. and tinct. opii ʒiss. in four doses; the ingredients in this compound fulfil many different indications.

The introduction of the finger of the patient into the rectum is recommended by an old and experienced practitioner, as an excellent means of controlling tenesmus. I have never seen it tried.

Passing from the province of medicine proper to that of surgery, a brief notice of wounds will close this paper.

Notwithstanding the many untoward circumstances which surrounded us, such as the drenching rains which almost universally succeed battles, vicissitudes of temperature, inadequate protection and almost entire want of hospital accommodation, in which our army is radically deficient, as well as in medicines and instruments, which had prepared us for the worst, I am happy to say that all ordinary wounds in sound constitutions healed as promptly and kindly as could have been desired, though in broken down and weak constitutions I think there was probably more sloughing and that convalescence was not as speedy as it would have been under more favorable circumstances. A fact which I may as well notice here is the remarkable rapidity with which maggots were generated. Wounds dressed on one morning were found on the succeeding to contain great numbers of these animals. If a dressing was allowed to remain on, or great care was not taken in cleansing the part, in 48 or 56 hours they were found collected in incredible quantities, and of enormous size, frequently as large as a small goose quill and from 3 to 9 lines in length. They were productive of no little pain from the irritation they produced in the parts, their tendency to burrow between divided muscles, and the great horror manifested by patients at their presence. As to their origin, there was much diversity of opinion. That they were produced by actual deposition from the fly, was hardly probable, since there were very few flies of this kind about the tents, and the wounds were entirely protected from their contact in many instances in which they occurred. It was held by some that the egg of the fly had been deposited in the dressings previously to their application; that this was not the fact, I am satisfied, since scalding the dressings, saturating them with turpentine and such other alternatives did not prevent their production; my own opinion is, thatthey were of spontaneous generation, of which, I think, I have abundant evidence. It not unfrequently became very difficult to to remove them from extensive wounds, from their tendency to burrow, especially when they took an upward direction, in which the means used for their destruction could not follow them. The means employed for this purpose were turpentine, chloride of sodium or lime, creosote, &c.; these generally succeeded in destroying one growth, but did not prevent its speedy replacement by another. From their position, I have frequently been compelled to remove them with instruments.

Gun shot wounds are of great extent and resemblance to almost every other variety of wounds. They resemble punctured wounds in progressing in the line of the force of the wounding body, are always more or less contused at the entrance, and lacerated at the exit, though there are sometimes exceptions to this, and are more commonly complicated by the presence of foreign bodies than all others combined. Great care should always be taken in examining such wounds. If there should be no foreign matter in sight as will frequently be the case, and the track of the wound be obscure, a probe should be carefully used; this will not unfrequently detect the foreign matter and enable you to remove it; much injury may result from a neglect of this important particular. Lead is probably the least irritating of all foreign bodies with which wounds are likely to be complicated. Should a ball therefore be deep seated or imbedded in bone or tendon, so as to offer much difficulty to its removal, it should be permitted to remain; nature remedies such evils by encysting. Pieces of clothing, patching of balls, hair, &c., are very rarely encysted; if they be not removed and sloughing set in you must cautiously watch for them; all attempts at curing a wound when thus complicated will prove abortive and fruitless. Extensive and dangerous abscesses are not unfrequently the consequences. There is rarely much hemorrhage in these wounds, the arteries seem enabled to slip by the offending body, which is generally of globular form. The general indications of treatment are perfectly plain, the same as in wounds of any description. The cold water dressing for the first few days is an excellent application, especially if too much inflammation be suspected. Nitric acid lotions and stimulating poultices, hasten the detachment of sloughs. A most excellent ointment is composed of rhubarb ʒss., opium gr. xv. and simple cerate an ounce; this fulfils all the indications—astringes, allays pain, and affords sufficient stimulation. A bandage, carried from the extremity of the injured limb above the seat of injury, is a valuable means for controlling muscular action, which is always an important item. Should too much constitutional irritation exist, purgatives leeching or venesection, will generally control it. Should a tonic treatment be required, which is most generally the case in extensive wounds, (though they are both sometimes necessary during the course of the cure) then alcohol is indispensable; quinine and iron in its various preparations are also precious remedies. The different bitters are also of much value, if the preceding remedies cannot be procured.

*Amputation.*—This important operation is too often heedlessly performed in the army. Our surgeons, relying on British authority, amputate when there still remain many chances for the recovery of the limb. The case may have been different with the half starved, dilapidated British soldier, to what it is with our robust, healthy, well-fed soldiery. Secondary amputations in Europe may have been followed by fatal consequences, but in this country I am convinced there is but little more risk in trusting a good constitution to a secondary than to a primary operation. Therefore, I say that no surgeon is justifiable in depriving the patient of so important a member, by a primary operation, when there is a possibility of recovery, if there be left to him the alternative of a secondary operation. This may be done, I repeat, only when from the position or extent of the wound a secondary operation would be inadmissible. Of 23 operations, which I witnessed in Mexico, six were secondary; of these only one died; the remaining 17 were primary, and three died; which, though on a small scale, shews the mortality to be nearly equal. The most evil consequences which I have witnessed to arise from secondary operations, were that the convalescence was slower and the drain upon the system very much greater. I will relate two or three cases in which amputation (primary) was decided upon, all of which recovered.

A. T., a privte in the 1st regiment of Mississippi volunteers, received an extensive wound in the knee, destroying the patella, a large portion of the inner condyle of the femur, and exposing the joint to a fearful extent; he refused to submit to an operation, and when I last saw him he was going about on one crutch. A private in the 1st Tennessee regiment had a compound comminuted wound of the radius and ulna, made by an escopet ball; recovery seemed almost impossible; he also refused to submit to an operation; he was so far convalescent when I last saw him, as to be able to use his hand in eating, washing his face, &c. The next, a very interesting case in more points than one, was that of L. Y. of our own regiment: he received an extensive contused and lacerated wound of the thigh, destroying all the muscles on the external and posterior region of the thigh, also some of the contiguous on the anterior portion, which exhibited when cleaned off a wound extending from the trochanter major to within three inches of the knee, and encircling about two-thirds of the limb. The patient was one of weak constitution and at the time somewhat emaciated from the diseases of the country (diar. and int. fever). The bone was not injured though denuded for three inches above the middle of the wound. Amputation at the hip joint was decided upon by almost every one who saw him. Dr. C. rather dissented, though probably, more from the knowledge of the patient's constitution and supposed inability to stand an operation, than from a hope of saving the limb or life of the patient. The operation was not performed. Dr. C. was taken ill and there were no instruments suited to the operation. The treatment was tonic from the start, the sloughing process went on slowly. Required constant stimulating poultices and lotions—a healthy ulcer of the dimensions given above was finally presented, the granulating process went on tolerably well. This was one of the cases in which maggots were so abundantly generated. During its course the case was complicated with a masked intermittent fever, which yielded to quinine which was after this time continued as a tonic. The case was also complicated with erysipelas, which was controlled by two applications of iodine. During the existence of these complications the ulcer assumed that peculiar glazed appearance which is so peculiar to ulcers when they cease to secrete. The tender cicatrice also ulcerated during these complications. The patient is now enabled to go about with a cane—the cicatrice is very tender.

February, 1848.

